# Whole-exome sequencing and bioinformatics analysis of a case of non-alpha-fetoprotein-elevated lung hepatoid adenocarcinoma

**DOI:** 10.3389/fphar.2022.945038

**Published:** 2022-08-26

**Authors:** Yao Yao, Xiaojiao Guan, Guangyao Bao, Jie Liang, Tian Li, Xinwen Zhong

**Affiliations:** ^1^ Department of Thoracic Surgery, First Affiliated Hospital, China Medical University, Shenyang, China; ^2^ Department of Pathology, Second Affiliated Hospital, China Medical University, Shenyang, China; ^3^ School of Basic Medicine, Fourth Military Medical University, Xi’an, China

**Keywords:** hepatoid adenocarcinoma of the lung, whole-exome sequencing, alpha-fetoprotein, lung cancer, clinical research

## Abstract

Hepatoid adenocarcinoma of the lung (HAL) is an exceptionally rare malignant tumor with prominent hepatocellular carcinoma (HCC)-like characteristics in organs or tissues outside the liver, while there is no tumor in the liver. Most HAL cases have various degrees of serum alpha-fetoprotein (AFP) levels and exhibit a similar origin and clonal evolution process to HCC. We studied a case of HAL without elevating the AFP level by performing whole-exome sequencing (WES) and bioinformatics analyses after surgical resection. Our results showed mutations in two driver genes, NLRP3 and PBX1, and we identified HNRNPR, TP73, CFAP57, COL11A1, RUSC1, SLC6A9, DISC1, NBPF26, and OR10K1 as potential driver mutation genes in HAL. In addition, 76 significantly mutated genes (SMG) were identified after the statistical test of each mutation type on genes.

## Introduction

Hepatoid adenocarcinoma (HAC), an exceptionally rare malignancy with prominent HCC-like characteristics in extra-hepatic organs or tissues, has been discovered in various organs, including the ovary (Mazouz et al., 2015), lung (Lin et al., 2013; Su et al., 2013; Shi et al., 2019), uterus (Kato et al., 2007), pancreas (Gardiner et al., 1992), and other sites. HAL occurs in approximately 5% of HAC cases (Haninger et al., 2014), and HAL was first reported by Ishikura et al. (1990). Although this rare disease has been identified for more than 30 years, the origin of HAC is still controversial to this date. During embryonic development, some organs like the liver, lung, stomach, and esophagus are diversified from the primitive foregut. Several abnormal differentiation processes may contribute to the development of HAC in these regions (Nakashima et al., 1987).

HAL cases are extremely rare, with less than 60 cases reported in the literature in the last 40 years (Hou et al., 2021). Usually, HAL patients exhibit chest and back pain, shoulder shooting pain, cough and breathlessness, shortness of breath, nausea, and weight loss (Sun et al., 2016). These symptoms are nonspecific and basically the same as those of other types of lung cancer, thus making accurate and timely diagnosis difficult. The main antidiastole for HAL is HCC metastases and non-small cell lung cancer. Collecting data on the patient’s medical history and clinical presentation is critical to the diagnosis as HCC often occurs in cirrhosis associated with hepatitis virus infection and alcohol abuse. Meanwhile, it is insufficient to use computed tomography (CT) images alone for diagnosis; cytomorphological and immunohistochemical techniques are necessary to confirm the diagnosis (Terracciano et al., 2003). According to the literature, most HAL tumors occur mainly in the upper lobes, usually near the large blood vessels in the chest wall or mediastinal pleura, while a small proportion of HAL tumors occur in the other lobes. The incidence of HAL is much higher in middle-aged and older male smokers than in women. Because of the nonspecific symptoms and difficulty in diagnosis, most patients are diagnosed with HAL after the disease has progressed to an intermediate or advanced stage (Zhuansun et al., 2021).

A significant increase of alpha-fetoprotein (AFP) in tissues or serum is common in most HAL cases (Ishikura et al., 1990; Grossman et al., 2016). By comparison, HAL without elevated AFP levels is more challenging to diagnose and treat owing to the scarcity of clinical cases and lack of evidence-based medicine (Sun et al., 2016).

The prognosis of HAL is worse than that of other types of lung tumors; the 5-year survival rate is only 8.0%, and the 2-year survival rate is 35.3% (Hou et al., 2021). At present, the understanding of HAL is still insufficient, and the etiology and underlying mechanism of such patients are still unclear; so far, there is no unified diagnostic standard, treatment, or prevention strategy to guide clinical practice. Surgical resection, chemotherapy, and/or radiotherapy are the most common treatments for this disease (Qian et al., 2016), and recently, molecular targeted therapy has shown great promise in treating HAL (Khozin et al., 2012). Therefore, this project provides a new basis for molecular diagnosis and targeted therapy of HAL through whole-exome sequencing (WES) and bioinformatics analysis.

## Materials and methods

### Sample

One patient, who was diagnosed with HAL in the First Affiliated Hospital of China Medical University, was enrolled in the study. Tumor and paracancerous tissues were rapidly frozen during surgery and saved in liquid nitrogen.

### DNA extraction and sequencing

DNA fragmentation was performed by NEBNext dsDNA Fragmentase (NEB, Ipswich, MA, United States), and the DNA fragments were then subjected to end-repair. In the next step, these DNA fragments were hydrolyzed and mixed with RNA library decoys and streptavidin-coated magnetic beads, followed by bead capturing, washing the beads, and RNA digestion, and finally, these DNA fragments were amplified to 150 bp. The cDNA/DNA/small RNA libraries were sequenced on the Illumina sequencing platform X Ten system (Illumina, San Diego, CA, United States) by Gene Denovo Biotechnology Co., Ltd. (Guangzhou, China).

### Quality control

Quality trimming is a very important step to guarantee high confidence in variant calling. Fastp (Chen et al., 2018) was used to complete the quality trimming. The filtering criteria were: 1) unidentified nucleotides (N) in the reads >10%; 2) the number of bases >50% of the reads with a phred quality score ≤20; 3) reads aligned to the barcode adapter. If the reads met one of these three criteria, the reads would be cleared.

### Variant identification and annotation

For mapping the valid sequencing reads to the reference human genome (GRCh38) and to identify SNVs and indels, the Burrows–Wheeler Aligner (BWA) (Li and Durbin, 2009) was used. Duplicate reads were marked and realigned using the Picard suite. The Genome Analysis Toolkit (GATK) (McKenna et al., 2010) Unified Genotyper was used to find variant sites on the genome, including SNV and small fragments of indels, and the base quality score recalibration. The software tool, ANNOVAR (Wang et al., 2010), was used to align and annotate SNVs or indels to the following databases: 1000 Genomes Project, HAMAP, ESP6500, dbSNP, and Kaviar.

### Mutation deleteriousness prediction

SIFT (Ng and Henikoff, 2003), PolyPhen-2 (Adzhubei et al., 2010), MutationTaster (Schwarz et al., 2010), and PROVEAN (Choi et al., 2012) were used to predict whether SNVs and indels lead to changes in protein structure and function.

### Driver gene identification

Driver mutation genes were identified by MuSiC (Dees et al., 2012) and OncodriveCLUST (Tamborero et al., 2013a). Comprehensively considering the preference of driver mutations in the locus distribution to form mutation clusters, we used synonymous mutations to construct a background mutation rate model with the characteristics of an unbiased distribution to find possible driver mutations by OncodriveCLUST software. MuSiC software was used to find genes with higher mutation frequency in tumor samples than control samples, and convolution tests were performed for each mutation type. The cutoff of MuSiC is FDR, 0.01 and that of OncodriveCLUST is FDR, 0.05.

### Gene functional enrichment analyses

We used the clusterProfiler package on R- 4.1.2 to map the significantly mutated genes to each term of the GO database (http://www.geneontology.org/) and each pathway of the Kyoto Encyclopedia of Genes and Genomes (KEGG) database and calculated the number of genes in each term and pathway, so as to obtain a list of genes with a certain GO function and pathway of KEGG. A hypergeometric test was then applied to find GO terms and KEGG pathways that were significantly enriched in genes compared to the whole-genome background. Modified Fisher’s exact test was applied, and *p* < 0.05 was considered a statistically significant difference.

### Structural variation identification and analyses

SV types include translocations, inversions, and insertion events, and SVs were determined using CREST (1.0) software (Wang et al., 2011). Copy number variants (CNVs) were identified using VarScan2 (Kadalayil et al., 2015) with the following parameters: Phred base quality ≥20 and minimum coverage ≥20. Recurrent somatic CNA identification was conducted by GISTIC (Mermel et al., 2011), and CNVs were classified using Control-FREEC (10.4) (Boeva et al., 2012).

## Results

### Case presentation

**FIGURE 1:**
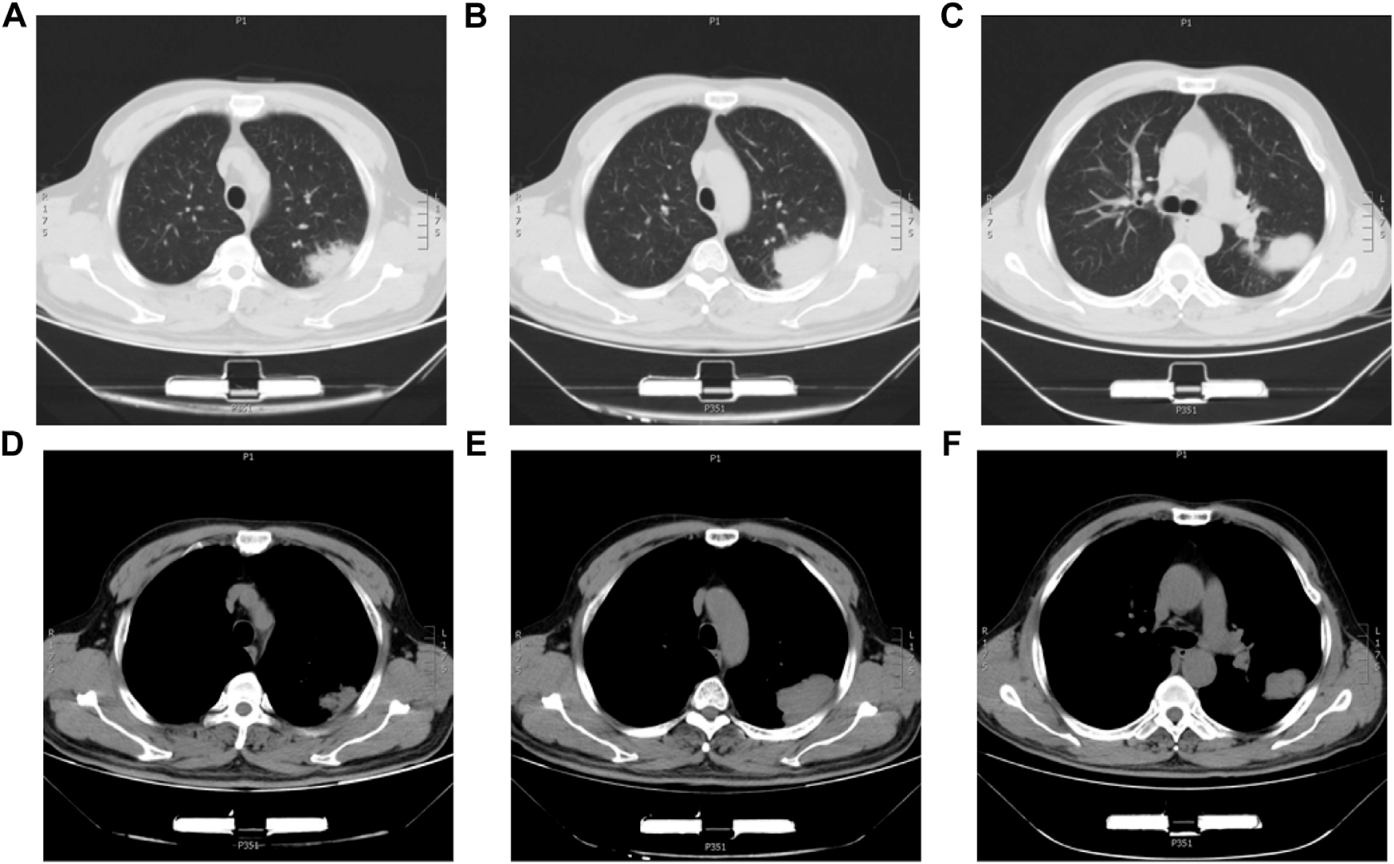
Chest CT images of the HAL patient. **(A,B,C)** CT images of the lung window with a lung mass in the left upper lobe. **(D,E,F)** CT images of the mediastinum window with a lung mass in the left upper lobe.

The 63-year-old male patient was admitted to the First Affiliated Hospital of China Medical University on 5 September 2018, with intermittent dyspnea and occasional chest pain. The chest-CT scan showed a 7.5*5.5 cm^2^ mass in the left upper lung with local pleural wrinkling and slightly enlarged lymph nodes in the mediastinum (Figure 1). Before the operation, no metastasis was found in brain CT or bone nuclide scan, while serum carcinoma embryonic antigen (CEA) and neuron-specific enolase (NSE) were slightly elevated, and AFP was in the normal range. On 19 September 2018, the patient underwent left upper lobe resection and mediastinal lymph node dissection. Referring to the preoperative abdominal B-ultrasound examination and postoperative abdominal B-ultrasound examination, 3 months after the surgery, there was no obvious abnormality in the liver, and the patient’s status was average with no history of hepatitis, alcohol abuse, exposure to radiation, or toxins. Meanwhile, he had no symptoms of liver cirrhosis such as spider-burst, liver palms, or hepatojugular reflex. We measured the patient’s plasma AFP levels 4 days before surgery, 1 day after surgery, and 3 months after surgery. The results of AFP were 11.02 ng/ml, 13.11 ng/ml, and 9.21 ng/ml, respectively. Given the patient’s symptoms, personal history, physical examination, preoperative examination, and immunohistochemical staining results of postoperative pathology, the patient was diagnosed with HAL. Meanwhile, there was no metastatic lesion in the mediastinal lymph nodes or adjacent tissue or organs; thus, the post-operative staging is T4N0M0. The patient recovered well after surgery and was discharged from the hospital one week later. Subsequently, a 300 gene-panel genomic testing was conducted, which contained targeted drug-related genes and chemotherapy drug-related genes. Nevertheless, no targeted drug-matched gene mutation was found in the testing. The patient did not receive radiotherapy or chemotherapy after surgery and died of an accidental cerebral hemorrhage six months later.

### Histological and immunohistochemical analysis

**FIGURE 2:**
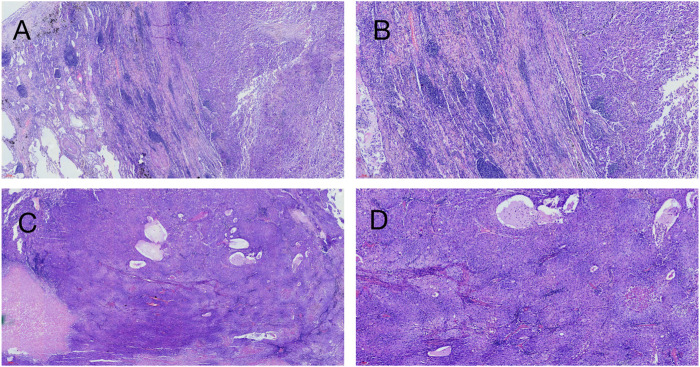
Hematoxylin-eosin staining on patient’s tumor tissue. **(A)** ( × 40, 200 µm), **(B)** ( × 100, 100 µm) Left side of the picture is the lung tissue, and alveolar fusion and inflammatory cell infiltration can be seen; the right side is a large tumor tissue, and fibrous tissue proliferation and inflammatory cell infiltration can be seen around the tumor tissue. **(C)** ( × 40, 300 µm), **(D)** ( × 100, 100 µm) Tumor tissue is distributed in the form of beams and nests, with abundant cytoplasm, similar to hepatocellular carcinoma.

**FIGURE 3:**
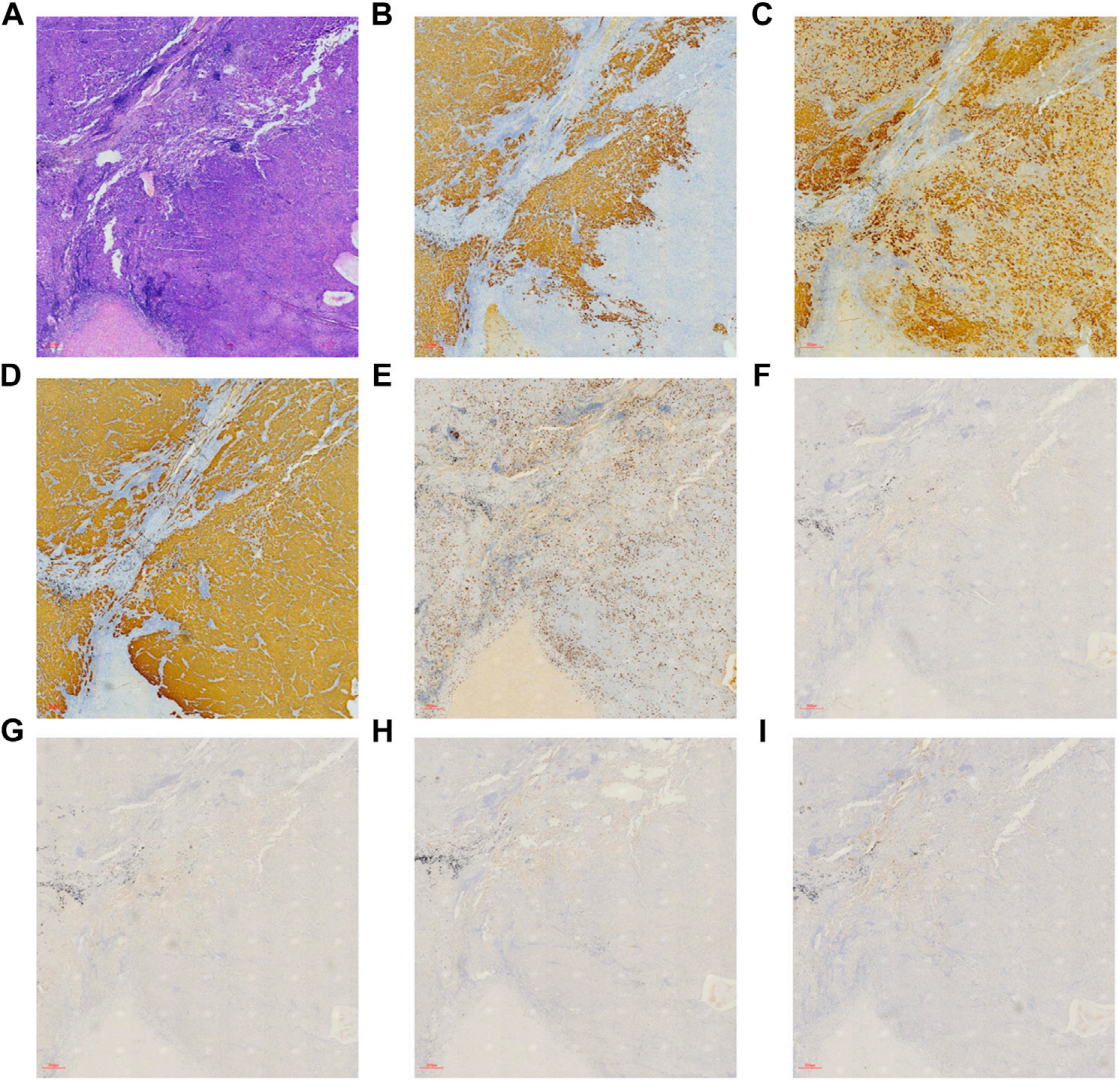
Immunohistochemical and hematoxylin-eosin staining on patient’s tumor tissue ( × 40, 300 µm). **(A)** HE staining, **(B)** hepatocyte (+), **(C)** glypican-3 (+), **(D)** CK (+), **(E)** Ki-67 (30% +), **(F)** P40 (−), **(G)** P63 (−), **(H)** TTF-1 (−), and **(I)** napsin-A (−).

Hematoxylin-eosin (HE) staining of tumor tissue suggested that the typical cell clusters were flaky, trabecular, and densely arranged; the nuclei were large and deeply stained, and the nucleoplasm ratio was imbalanced. Additionally, alveolar expansion with fracture and fusion was found in the lung tissue (Figure 2). Immunohistochemistry staining demonstrated hepatocyte (+), glypican-3 (+), cytokeratin (CK) (+), Ki-67 (30% +), P40 (−), P63 (−), thyroid transcription factor-1 (TTF-1) (−), and napsin-A (−) (Figure 3).

### Whole-exome sequencing and analysis of somatic mutations

**FIGURE 4:**
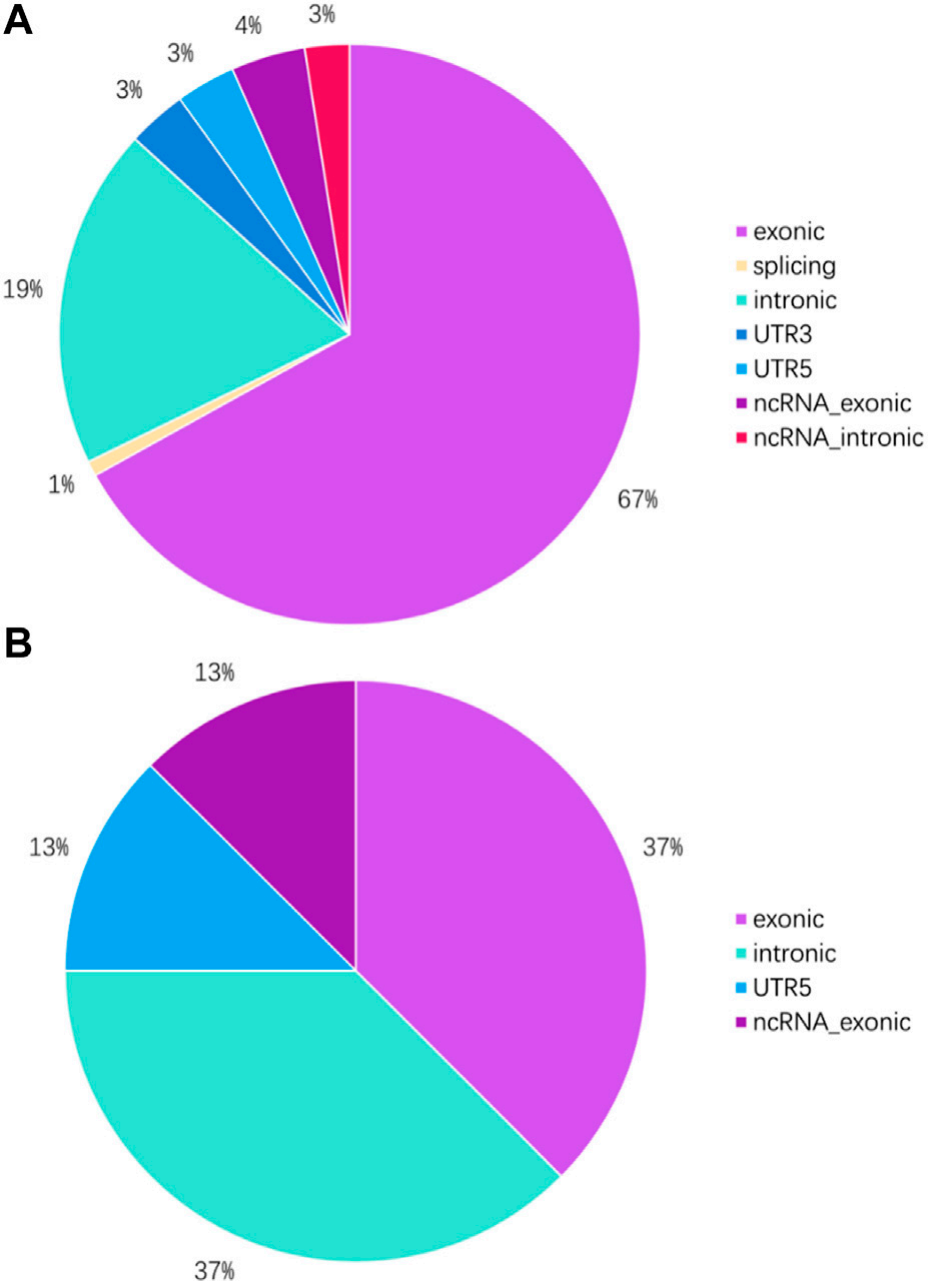
Genome location of SNVs and indels in tumor tissue. **(A)** Proportion of SNVs in different parts of the genome. **(B)** Proportion of indels in different parts of the genome.

**FIGURE 5:**
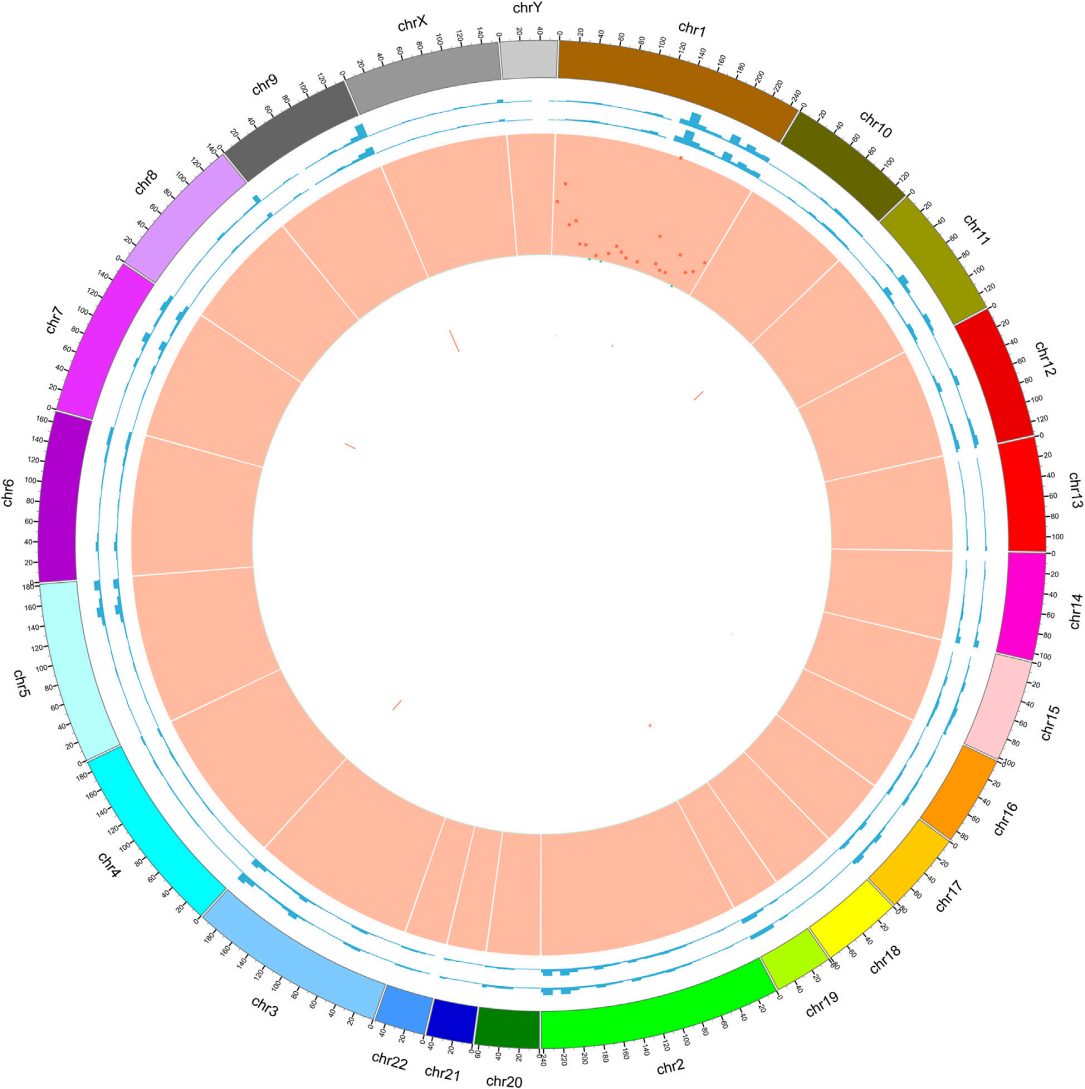
Circle graph of somatic mutations found in tumor samples. Circle 1: outer frame of the chromosome; Circle 2: sequencing coverage map of tumor samples; Circle 3: sequencing coverage map of normal samples; Circle 4: green dots indicate the density of SNP indel; Circle 5: CNV results, red indicates the copy number increase; Circle 6: CNV results, blue indicates copy number deletion; we found that by comparing with paracancerous samples, the differential mutations in tumor samples were mainly located on chromosome 1.

To investigate the mutation in this case of HAL, we performed whole-exome sequencing of the tumor tissue. After sequencing and clean read filtering, 122 somatic SNVs and 3 somatic indels were detected using MuTect (Cibulskis et al., 2013), most of which were situated in the exonic parts of the genome (Figure 4). Also, we used VarScan2 (28) to filtrate the somatic CNVs. Correlation analysis revealed that most somatic mutations clustered in chromosome 1(Figure 5). Among the somatic mutations detected in the tumor samples, two alternations were found in the coding region of NLRP3, including one synonymous mutation (c.1299C>A) and one nonsynonymous mutation (c.1300 C>A). Also, one nonsynonymous mutation (c.731C>T) was detected in the exonic region of PBX1, after searching reported driver gene databases, including Cancer Gene Census, MDG125 (Vogelstein et al., 2013), SMG127 (Kandoth et al., 2013), and CDG291 (Tamborero et al., 2013b). The synonymous mutation (c.1299C>A) in NLRP3 was archived in the COSMIC database from a lung adenocarcinoma tumor sample (TCGA-50-5941-01), and the nonsynonymous mutations of neither NLRP3 nor PBX1 were archived in dbSNP, 1,000 Genomes Project, ExAC database, and COSMIC. So, we annotated the mutations with MutationTaster, SIFT, PolyPhen2, and PROVEAN to predict whether these mutations are detrimental to protein function. The PolyPhen2 predicted the variant (c.1300C>A) to be benign with a score of 0.016 and the score of the variant (c.731C>T) was 0.997, so it was determined to be probably damaging. Meanwhile, PROVEAN predicted the variant (c.1300C>A) to be neutral with a score of −1.75 and the variant (c.731C>T) to be deleterious with a score of −3.69. Although the *NLRP3* variants (c.1300C>A and c.1299C>A) and the *PBX1* variant (c.731C>T) have been identified, there are no recommended therapies for diseases caused by these genetic mutations at present.

**FIGURE 6:**
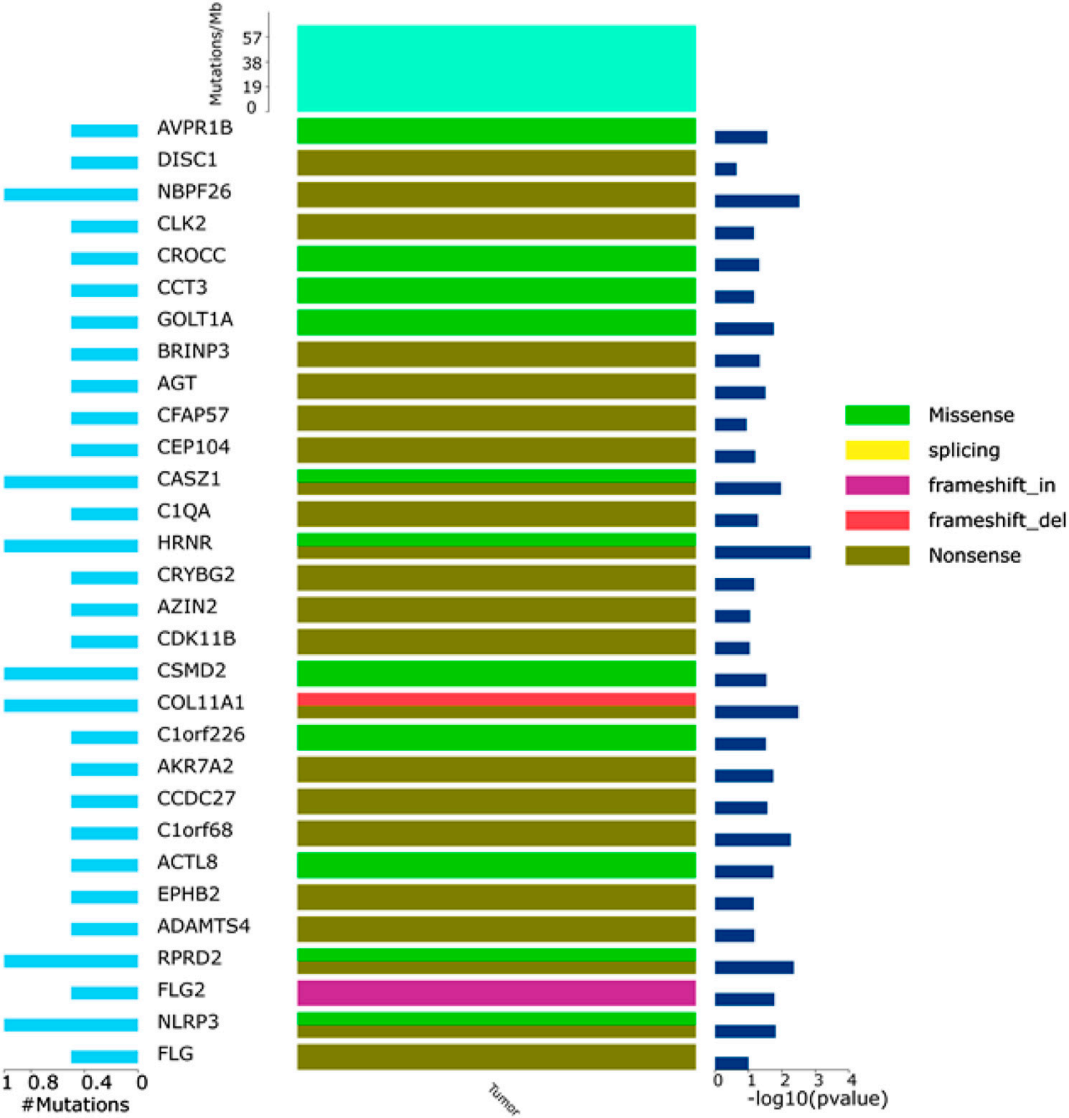
General map of high-frequency mutations. Left: percentage of genetic mutations; right: high-frequency mutant gene significance; top: mutation frequency of samples; middle: mutation types, including I. missense (green), II. splicing (yellow), III. frameshift_in (purple), IV. frameshift_del (red), and V. nonsense.

**FIGURE 7:**
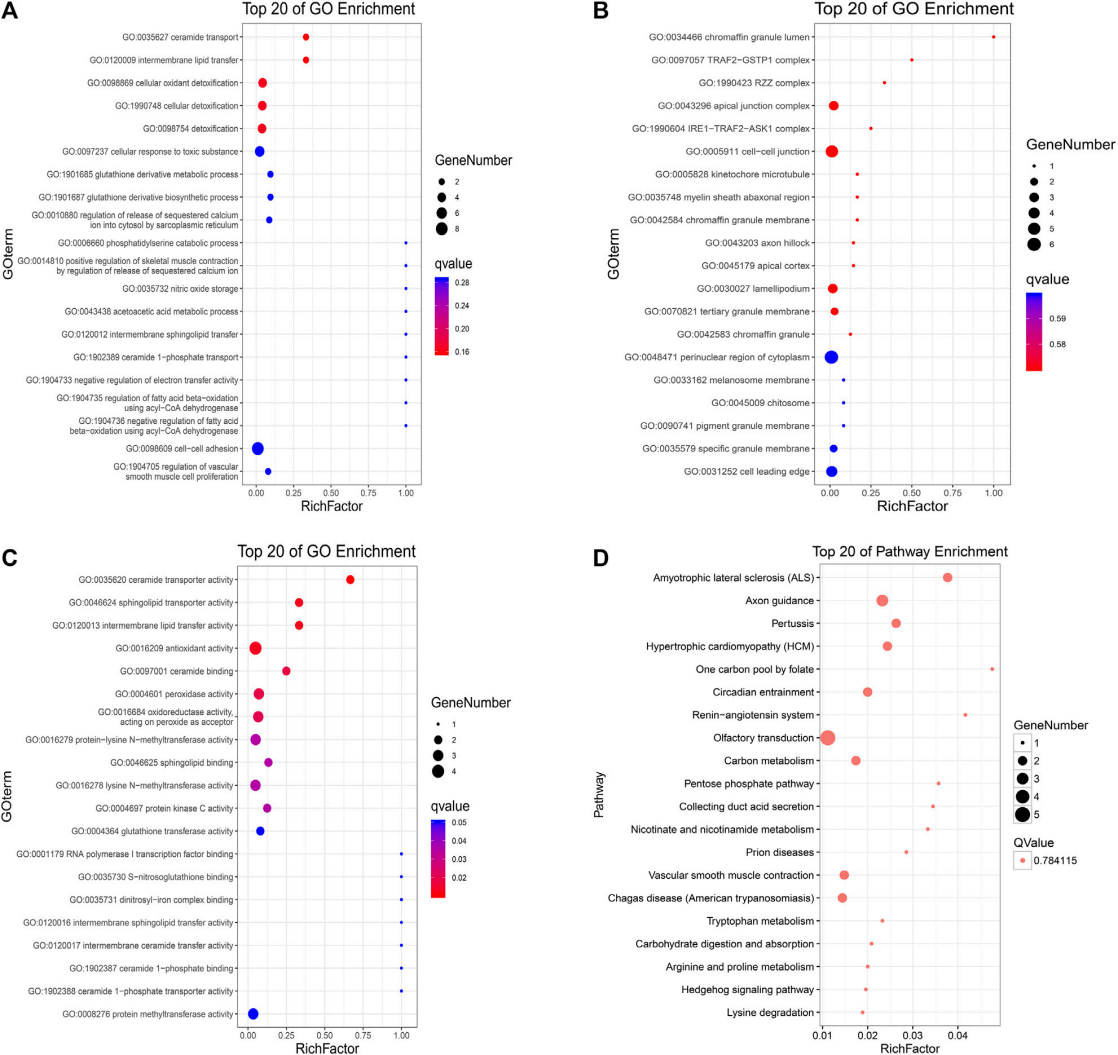
KEGG and GO analyses of identified high-frequency mutant genes. Gene Ontology **(A)** biological process, **(B)** cellular component, and **(C)** molecular function; *X*-axis: the ratio of enriched genes to the total genes; *Y*-axis: GO terms. The dot size represents the number of enriched genes, and the color represents the *p*-value. **(D)** KEGG pathway analysis; *X*-axis: the ratio of enriched genes to the total genes; *Y*-axis: the terms of pathways. The dot size represents the number of enriched genes, and the *Q*-value is 0.784115.

To further verify those cancer-related mutations, we used MuSiC(25) to seek significantly mutated genes in cancer samples, and simultaneously, all the somatic alternations were statistically tested by the convolution test (Figure 6). GO and KEGG interaction analyses were conducted among the significantly mutated genes, which are shown in Figure 7. The results showed that, for molecular function, these significantly mutated genes (SMGs) mainly participated in ceramide transporter activity, sphingolipid transporter activity, intermembrane lipid transfer activity, antioxidant activity, ceramide binding, and peroxidase activity (all *p*-adjusted <0.05). Additionally, “amyotrophic lateral sclerosis (ALS)” (*p*-adjusted = 0.012624) and “axon guidance” (*p*-adjusted = 0.032708) are the KEGG pathways that were mainly enriched by SMGs.

## Discussion

### Interpretation

The incidence of HAL is extremely low, accounting for only about 5% of HAC. Among them, smoking men above middle age are the most commonly affected individuals, and most patients have elevated AFP levels. In recent years, some patients without elevated AFP levels have occasionally appeared. Most of the lesions in patients with primary HAL appear in the upper lobe of the lung, resulting in the symptoms and imaging findings of the patients being very similar to those of other types of lung cancer, but HE and immunohistochemical staining of the tumor pathology showed HCC-like features (Zhuansun et al., 2021). Therefore, to avoid misdiagnosis, CT examination and HE and immunohistochemical staining should be conducted together to help in the diagnosis (Terracciano et al., 2003). Ishikura et al. (1990) proposed two diagnostic criteria for HAL at that time, namely, typical acinar or papillary adenocarcinoma, most of which were like HCC, and either the expression of AFP was positive or there was a significant increase of AFP in the serum content. However, the aforementioned clinical diagnostic criteria are imperfect, and occasional cases of HAL without elevated AFP had been reported over the next few years. In the first case of HAL proposed by Haninger et al. (2014), there was no increase in AFP, and the pathological manifestations were tissue neuroendocrine carcinoma and signet ring cells. Therefore, Haninger redefined the criteria for HAL diagnosis. First, the tumor can be a typical acinar or papillary adenocarcinoma, signet ring cell carcinoma, or containing neuroendocrine function. Second, if it expresses markers of liver differentiation, the abnormality in the patient should be diagnosed as HAL, no matter the AFP level is elevated or not. A thorough review of the published cases showed that patients with early diagnosis of HAL had longer postoperative survival, and the current treatment for HAL is the same as that for other lung cancers; radiotherapy and chemotherapy are effective treatments for patients with advanced HAL (Zhuansun et al., 2021). However, owing to the lack of analysis of gene mutations in HAL cases, the progress of new gene-targeted therapy has been limited. Li et al. (2019) conducted whole-genome sequencing (WGS) in a HAL patient with elevated AFP and detected a FAT1 driver gene mutation. FAT1 is one of the common mutations in malignant tumors, which inhibits tumor growth through the activation signal of Hippo signaling (Li and Guan, 2021) and promotes tumor migration by inducing actin polymerization (Katoh, 2012; Coudray et al., 2018). However, there are no effective drugs against FAT1 mutation. Chen et al. (2019) reported a case of HAL harboring an EGFR mutation and being responsive to tyrosine kinase inhibitor (TKI) therapy (Harrison et al., 2020). Also, Chen et al. (2020) reported a case of HAL with a KRAS driver mutation, but due to the lack of effective drugs against KRAS mutations, the patient experienced fifth-grade pneumonia and died after 6 months of anti-PD-1 treatment. Sun et al. (2022) performed next-generation sequencing (NGS) on three primary HAL cases and one HAS-metastatic HAL case and detected TP53 mutations in all four patients, although TP53 is a particularly common driver mutation. There have been several tumor studies targeting p53 inactivation (Chen et al., 2021; Liu et al., 2022), but there are no effective therapeutic agents for TP53 mutations currently, with only three compounds having reached phase III clinical trials: the mutant p53-reactivating drugs, APR-246 and COTI-2, and the MDM2 antagonist idasanutlin (Duffy et al., 2022). Meanwhile, CDK8, CKDN2A, CSF1R, EPHA5, PKHD1, SMARCA4, and STK11 were detected as high-frequency mutations with a mutation rate of 50%. The expression of AFP in the immunohistochemistry of one out of four patients was also negative, and the gene mutations detected by sequencing were TP53, CDKN2A, STK11, and INPP4B, which were different from our sequencing results. In our study, only two nonsynonymous mutations, variant (c.1300C>A) in NLRP3 and variant (c.731C>T) in PBX1, were detected. NLRP3, the sensor component of the NLRP3 inflammasome, plays a crucial role in innate immunity and inflammation (Nakanishi et al., 2017). Inflammasomes can also induce pyroptosis, an inflammatory form of programmed cell death (Mitoma et al., 2013; Mishra et al., 2022). Hamarsheh summarized the therapeutic potential of targeting NLRP3 inflammasome in cancer (Hamarsheh and Zeiser, 2020). Moreover, some small-molecule compounds have anti-inflammatory effects that can be designed for drugs, such as thalidomide (Franks et al., 2004) and VX-765 (Wannamaker et al., 2007). PBX1 belongs to the PBX homeobox family of transcriptional factors. The *E2A-PBX1* fusion gene caused by t (McKenna et al., 2010; Mazouz et al., 2015) (q23; p13) can be found in many types of cancers, which makes it an attractive potential target for the design of new drugs (Mo et al., 2013).

**FIGURE 8:**
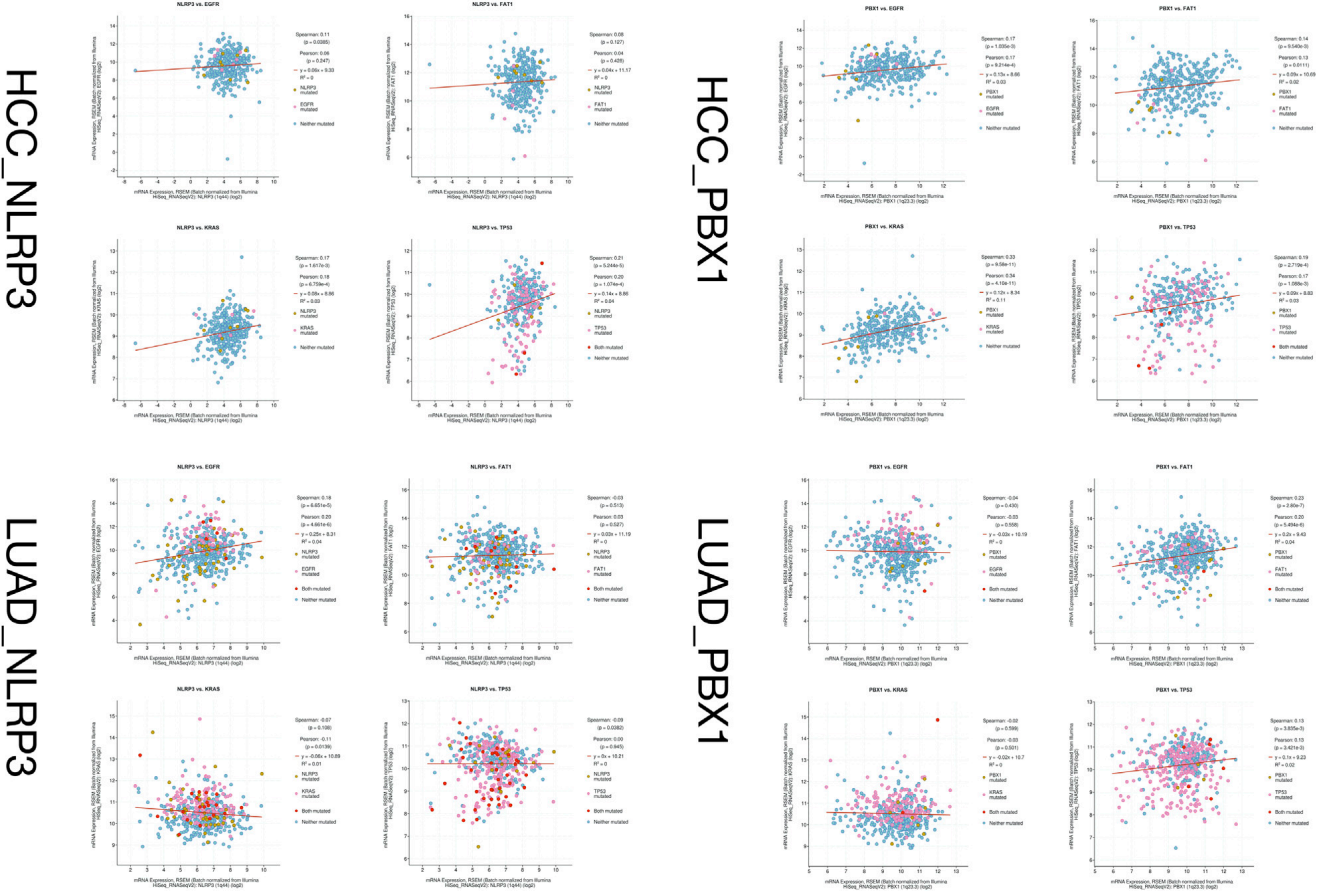
Co-expression information between mutated NLRP3, PBX1, FAT1, EGFR, KRAS, and TP53 in HCC and LUAD tissues.

Given the similar pathological features of HAL, hepatocellular carcinoma (HCC), and lung adenocarcinoma (LUAD), there may be similar disease progression among them. We compared the gene mutation information of NLRP3 and PBX1 in HCC (data from TCGA, PanCancer Atlas with 372 samples) and LUAD (data from TCGA, PanCancer Atlas with 566 samples) through the cBioPortal database (https://www.cbioportal.org/) and analyzed the relationship between HAL-related driver gene mutations. NLRP3 is altered in 11% of 566 cases in the mutation data from TCGA-LUAD samples and 2.2% of 366 cases in the mutation data from TCGA-HCC samples, and PBX1 is altered in 1.8% of 566 cases in the mutation data from TCGA-LUAD samples and 1.6% of 372 cases in the mutation data from TCGA-HCC samples. After comparing the co-expression information between mutated NLRP3, PBX1, FAT1, EGFR, KRAS, and TP53 in HCC and LUAD tissues through the cBioPortal database, we found that the samples with both NLRP3 and TP53 mutations were the most in LUAD (Figure 8).

Despite these studies showing that some gene mutations lead to the occurrence and development of HAL, its potential mechanism needs to be explored. Comprehensively considering the preference of driver mutations in the locus distribution to form mutation clusters, we used synonymous mutations to construct a background mutation rate model with the characteristics of an unbiased distribution to find possible driver mutations using OncodriveCLUST software. We identified HNRNPR, TP73, CFAP57, COL11A1, RUSC1, SLC6A9, DISC1, NBPF26, and OR10K1 as potential driver mutation genes in HAL.

HNRNPR was originally identified as a component of the heterogeneous nuclear ribonucleoproteins (hnRNPs) family, which plays a vital role in many aspects of (pre)mRNA processing (Dreyfuss et al., 2002). HNRNPR expression is altered in many malignancies, suggesting that it is associated with tumor formation (Pereira et al., 2017). The characteristics of HNRNPR in neurodevelopment have also been reported in many literature works (Dombert et al., 2014), which explain the occurrence of neuroendocrine differentiation in tumors of HAL. TP73 is a member of the TP53 family, mutations of which are found in many tumors and are associated with prognosis. TP73 can transform multiple variants with oncogenic and tumor-suppressive functions (Jost et al., 1997). Collagen type XI α1 (COL11A1), a minor fibrillar collagen, which plays a crucial role in cell proliferation, migration, and tumorigenesis of many malignancies, may be a valuable diagnostic marker for lung cancer. Smad signaling is activated by the overexpression of COL11A1 and promotes cell proliferation and migration (Shen et al., 2016). DISC1, an oncogene, activates the GSK3β/β-catenin signaling to promote NSCLC growth, so it could be designed to be a novel anti-NSCLC therapeutic target (Wang et al., 2017), which means that HAL has the same mutation gene and differentiation process as NSCLC does.

In this study, a comprehensive interaction analysis of GO and KEGG was performed among 76 significantly mutated genes to gain a more specific understanding of tumor genome mutation enrichment and biological functions. GO-cellular components revealed that these high-frequency mutated genes were mainly related to cell, cell part, and organelle. The GO-biological process showed these genes were related to cellular process and single-organism process, and GO-molecular function showed that these genes were related to binding function. Additionally, KEGG pathway enrichment revealed that the mutant genes mainly participated in “amyotrophic lateral sclerosis (ALS)” and “axon guidance” These findings suggest that these pathways that mutant gene aggregation enriched might play a vital role in the tumorigenesis process.

### Limitation and strength

This study has several limitations, especially the small size of the sample due to the rarity of HAL, and second, there were some false-negative results caused by the inadequate sequencing coverage inevitably, despite the sequencing depth and coverage of WES being much more improved than the Sanger sequencing. Finally, there is heterogeneity in different parts of the tumor, and the tissue obtained from the biopsy is so finite that it is impossible to cover the whole tumor genome. However, it was the first time that the WES was conducted on a non-AFP elevated HAL patient to explore the potential mechanisms of tumorigenesis. Meanwhile, the gene mutations are valuable to be the prospective candidates for gene-targeted therapy.

### Further clinical guidance

Cancer is caused by abnormal cell division and differentiation due to genetic mutation (Heng and Heng, 2022); therefore, inhibiting mutations in genes related to tumor formation is the most fundamental approach for treating cancer, and with the increasing maturity of next-generation sequencing technology, more and more gene-specific research studies are being conducted in cancer diagnosis and treatment. In this study, our goal was to identify rare deleterious variants and biological pathways of HAL mutant gene enrichment that will guide future genetic and functional studies on the pathogenesis of this rare malignancy.

In the past, cancer was classified according to its morphological characteristics. To date, understanding of cancer has reached the molecular level, and based on next-generation sequencing technology, individualized precision medicine is becoming more widespread. Therefore, it is necessary to recommend targeted drugs for patients with different genetic backgrounds.

## Conclusion

In summary, the rarity of HAL making large-scale trials difficult to organize, we used WES to reveal molecular patterns of HAL without an elevated AFP level. As we know, this is the first time that NLRP3 and PBX1 mutations were found in HAL without an elevated AFP level, which suggests the underlying mechanisms of tumorigenesis. Clinical treatment of HAL should not be based only on the treatments of common lung cancer or HCC; individualized and precise treatment should be implemented for its unique driver gene mutations, and our research reveals the potential mechanism of its development and provides a new potential target for treating this rare tumor.

## Data Availability

The datasets presented in this study can be found in online repositories. The names of the repository/repositories and accession number(s) can be found at: https://www.ncbi.nlm.nih.gov/, SUB11172444.
